# Comparing the Effectiveness of Platelet‐Rich Plasma Alone Versus Combined With Microneedles or Radiofrequency for Neck Wrinkle Treatment

**DOI:** 10.1111/jocd.16651

**Published:** 2024-11-06

**Authors:** Ke‐Cheng Li, Zi‐Zhe Lin, Zhi‐Dan Zhang, Shan Xie, Guang‐Hui Xie

**Affiliations:** ^1^ Department of Plastic Surgery The First Affiliated Hospital of Jinan University Guangzhou Guangdong China; ^2^ Shenzhen Longhua District Central Hospital Shenzhen Guangdong China

**Keywords:** microneedles, neck wrinkles, platelet‐rich plasma, radiofrequency

## Abstract

**Background:**

Neck wrinkles are a prominent characteristic of skin aging, with recent studies indicating that interventions such as platelet‐rich plasma (PRP), microneedling (MN), and radiofrequency (RF) can effectively rejuvenate aging skin.

**Aims:**

This study aims to assess and compare the efficacy of three treatment modalities in addressing neck wrinkles.

**Methods:**

Fifteen female participants with a neck Lemperle Wrinkle Assessment Scale (WAS) score of 3–4 were enrolled in a randomized clinical trial. The subjects were randomly assigned to three treatment groups: PRP injection, MN + PRP topical PRP application, and RF + PRP injection. They received treatment once a month for three consecutive months, and a clinical outcome evaluation was performed at 1 and 6 months after the final treatment.

**Results:**

The WAS scores and global aesthetic improvement score (GAIS) demonstrated notable enhancements 1 month postfinal treatment across all three groups, with a notably greater number of participants experiencing improved outcomes in the RF + PRP group 6 months posttreatment. Specifically, at the 1‐month follow‐up, the RF + PRP group exhibited a statistically significant enhancement in skin elasticity and collagen, surpassing the improvements observed in the other two groups (*p* < 0.05). No significant disparities in skin elasticity and collagen were detected in all groups prior to and after 6 months of treatment. Adverse events were mild and transient, such as redness, swelling, bruising, and pain.

**Conclusion:**

This study revealed that all the treatments can effectively improve neck wrinkles 1 month after the last treatment. A better therapeutic effect could be observed in the RF + PRP group compared with the other two groups at 6 months after the last treatment.

## 1 Introduction

The emergence of neck wrinkles, characterized by linear grooves in the anterior half of the neck skin, has garnered increased attention in recent years within the academic community. While skin aging is widely acknowledged as the primary cause of facial wrinkles, the formation and progression of neck wrinkles are recognized as a multifactorial process closely associated with skin aging, though not exclusively attributed to it [1]. Factors such as frequent tilting of the head to use electronic devices, prolonged periods of desk work, and obesity have also been identified as potential contributors to the development of neck wrinkles [2]. The presence of neck wrinkles can have a significant impact on both physical appearance and overall well‐being, including psychological health. Consequently, the increasing desire to address signs of neck aging has prompted the emergence of various neck rejuvenation treatments, including laser therapy, radiofrequency technology, botulinum toxin injections, soft tissue fillers, and biostimulator product injections [3, 4, 5, 6, 7].

Recent studies have demonstrated that platelet‐rich plasma (PRP) accelerates wound healing and promotes tissue regeneration through either topical application or direct injections [8, 9, 10]. PRP, an autologous biological product derived from blood, is rich in growth factors. Its accessibility, renewability, cost‐effectiveness, lack of ethical concerns, and suitability for individuals of all ages have led to a growing interest and utilization of PRP. RF technology has the ability to penetrate the epidermis and deliver a thermal stimulus to the dermis and subcutaneous tissues, leading to collagen denaturation and fibroblast stimulation for neocollagenesis, ultimately resulting in tissue tightening and wrinkle reduction. Microneedling (MN) is a minimally invasive therapeutic approach utilized for the treatment of various skin problems by creating controlled skin microinjuries to facilitate drug delivery or induce collagen synthesis and skin rejuvenation.

PRP can be effectively combined with other procedures such as MN and RF, to achieve enhanced outcomes [11]. However, there is a gap in the literature regarding the assessment of the effectiveness and safety of PRP treatment in conjunction with radiofrequency (RF) or MN for patients with neck wrinkles. This study aims to investigate the efficacy of PRP injection alone and in combination with MN or RF for improving neck wrinkles.

## 2 Materials and Methods

### 2.1 Subject Selection

**FIGURE 1:**
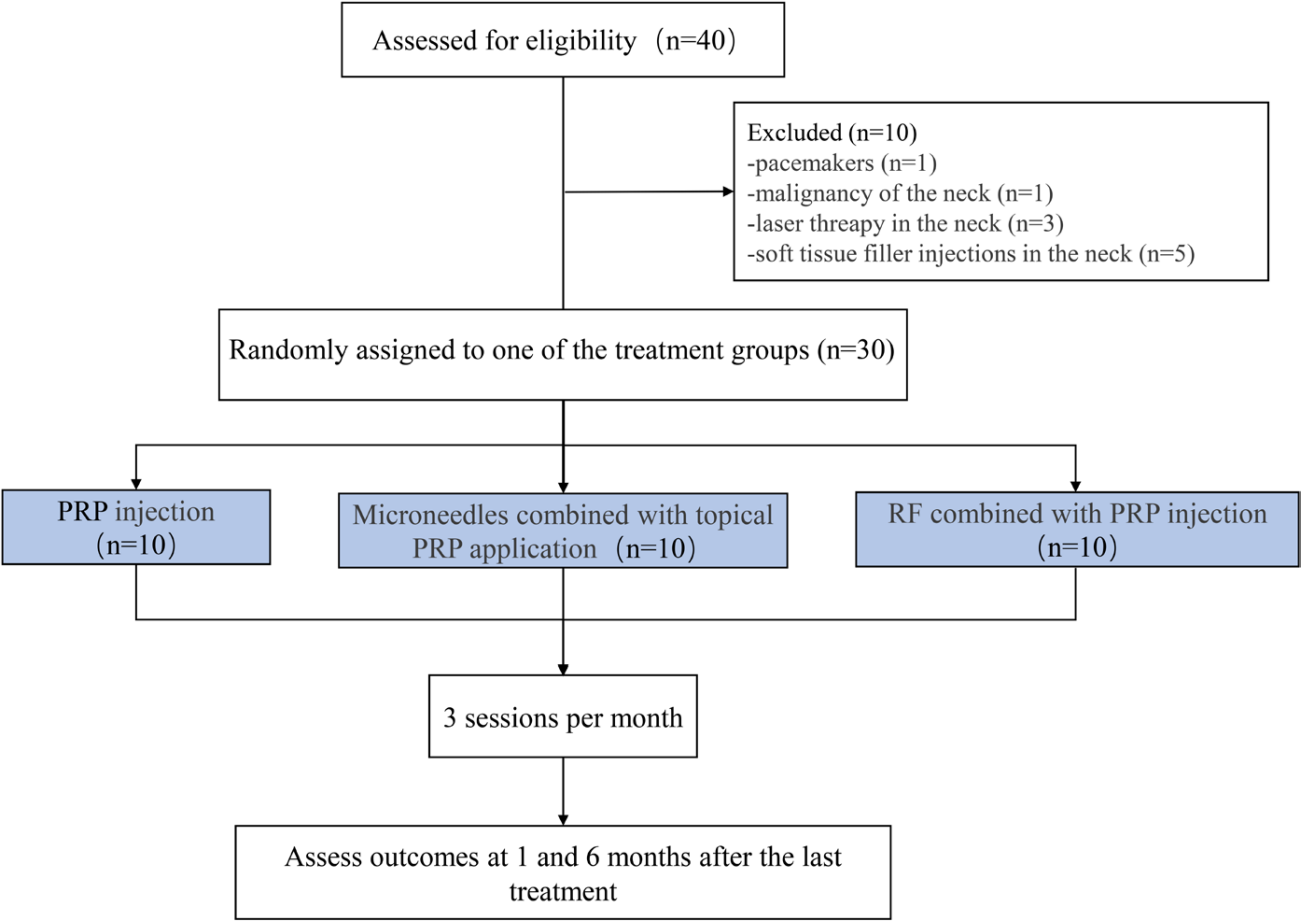
Flowchart showing the procedure for participating and assessment process in this study.

A total of 15 healthy female participants (mean age: 40 years, range: 36–49 years) with neck wrinkles rated as 3 or 4 according to the Lemperle Wrinkle Assessment Scale (WAS) [12] (Table 1), desiring a treatment to improve the appearance of neck wrinkles were recruited from August to October 2020. They were randomly assigned to one of three treatment groups, with five subjects per group. Group PRP was treated with PRP injection, while group MN + PRP was treated with microneedles combined with topical PRP application, and group RF + PRP was treated with RF combined with PRP injection. The flowchart of enrollment is presented in Figure 1.

**TABLE 1:** The Lemperle Wrinkle Assessment Scale.

Grade	Description
0	No wrinkles
1	Barely perceptible wrinkles
2	Shallow wrinkles
3	Moderately deep wrinkles
4	Deep wrinkles, well‐defined edges
5	Very deep wrinkles, redundant fold

Exclusion criteria included patients who were pregnant or breastfeeding, had pacemakers or defibrillators, exhibited coagulopathy, had an immunocompromised status, or had inflammation or malignancy of the neck. Additionally, individuals who had undergone soft tissue filler injections, any type of laser or RF, ultrasound treatment in the neck, or botulinum toxin injections into the platysma within 6 months prior to enrollment were also excluded from the study.

### 2.2 Photographic Methods

High‐resolution photographs were taken of all subjects before treatment and at each follow‐up visit, utilizing consistent camera equipment, lighting conditions, and distance from the subjects. Images of the subjects' necks were obtained with their chins elevated to the lower lip and the bilateral earlobe aligned in a straight orientation. Both frontal and 45° angle views of the neck were photographed in order to evaluate the presence of neck wrinkle.

### 2.3 Autologous PRP Preparation

Sixty milliliters of whole blood were collected from the antecubital vein of patients and stored in a vacuum blood collection tube containing 3.2% sodium citrate (Yangpu Medical Instruments Co. Ltd., Guangzhou, China). PRP was isolated using a two‐stage centrifugation process. Initially, the tubes were centrifuged at 300×*g* for 15 min at 25°C, resulting in the separation of blood into plasma, buffy coat, and red blood cells. Subsequently, the plasma and buffy coat were transferred to a new tube, and platelets were pelleted by centrifugation at 600×*g* for 15 min. The pelleted platelets were then resuspended in platelet‐poor plasma, yielding a final volume of 6 mL PRP. The number of platelets measured by the hematology analyzer and the platelet concentration in PRP was five times higher than that in the whole blood. Finally, prior to injection, PRP was mixed with 10% calcium gluconate at a ratio of 9:1 to activate the platelets.

### 2.4 Treatment

All patients' cervical skins were cleaned thoroughly, photographed prior to treatment, and anesthetized by applying compound lidocaine cream (2.5% lidocaine and 2.5% prilocaine; Tsinghua Tongfang Co., Beijing, China) for 1 h before treatment. After achieving satisfactory anesthesia, the neck skin was sterilized.

**FIGURE 2:**
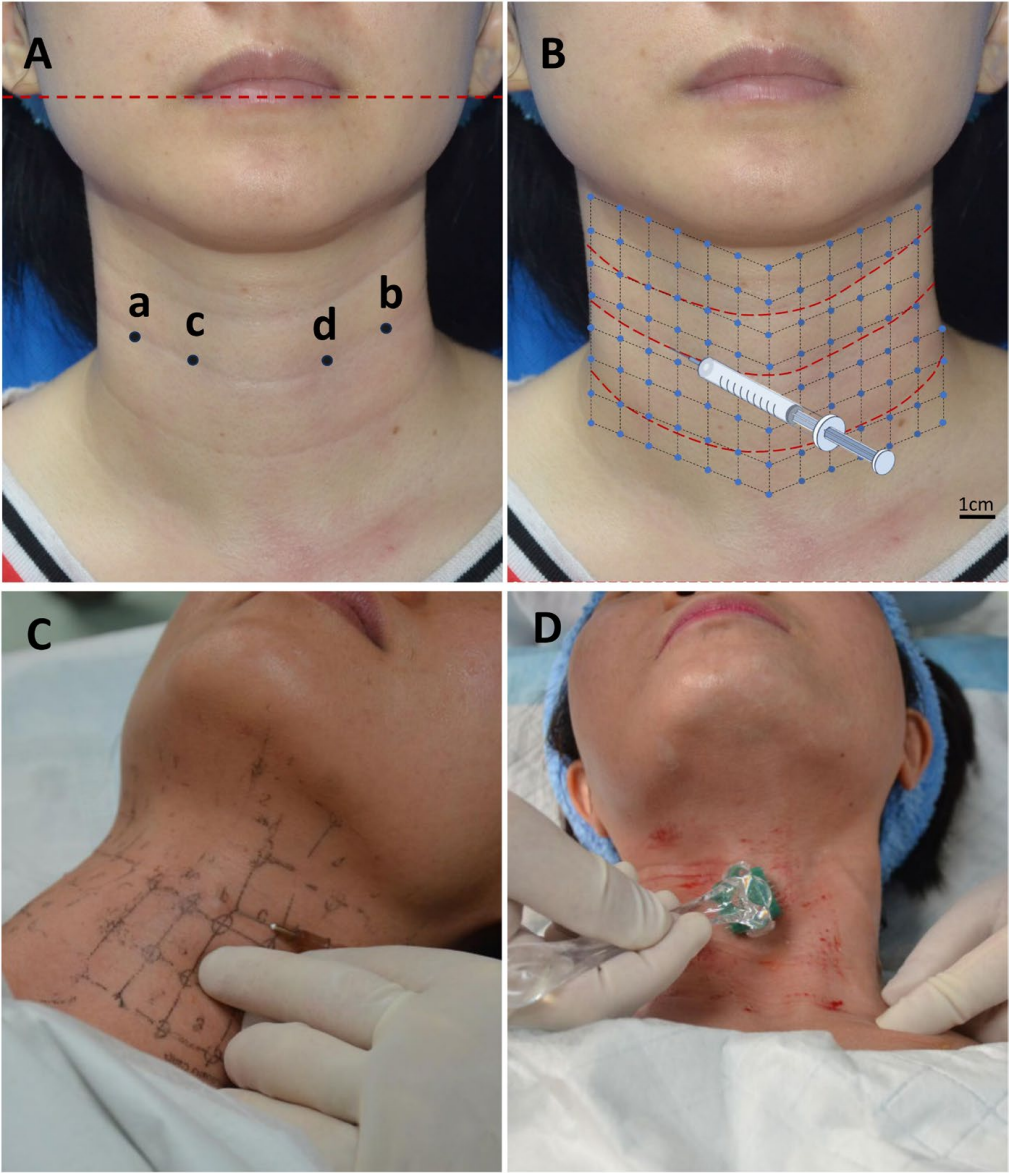
(A) Measurement points for the evaluation of collagen and elastin. The junction of the most prominent neck wrinkle and the bilateral anterior border of the sternocleidomastoid muscle (points a and b), the midpoint of the line between point a and the neck midline (point c), the midpoint of the line between point b and the neck midline (point d). (B) Injections were performed with simultaneous withdrawal of the needle along the horizontal wrinkles (red dashed line). Then, injections were spaced in intervals of about 1 cm on the neck and 0.05 mL PRP was injected per site (blue points). (C) Schematic view of PRP injections. (D) Schematic view of microneedling treatment.

Each group underwent monthly treatment for a duration of 3 months. Participants in the PRP group were administered PRP injections. First, the activated PRP was linearly injected into the dermal layer of the neck along the horizontal wrinkles. The injections were performed with concomitant needle retraction, delivering 0.1 mL of PRP per 1 cm of wrinkles. Subsequently, the injection sites were delineated at 1‐cm intervals on the neck, with 0.05 mL of PRP being injected at each site (Figure 2B,C).

The group PRP + MN underwent treatment involving MN in conjunction with the topical application of PRP. The MN procedure utilized a device equipped with 192 needles measuring 2 mm in length, which was rolled across the neck skin five times in horizontal, vertical, and diagonal directions with consistent and firm pressure. Following the completion of MN, PRP was promptly applied topically.

The Group PRP + RF received treatment with RF combined with PRP injection. Subjects with neck wrinkles underwent treatment with Accent Pro (Alma Lasers Ltd. Caesarea, Israel), a noninvasive unipolar RF skin tightening device. The RF parameters included a total energy, 30 kJ, a power range of 80–120 W, depths of 1.5 mm and 2.5 mm, and maintenance of epidermal skin temperature at 40°C–42°C. PRP injection was also performed immediately as described above after the RF treatment was complete.

### 2.5 Evaluation

#### 2.5.1 Neck Wrinkle Assessment

The evaluation of treatment outcomes was conducted by two impartial plastic surgeons who were blinded to the method of treatment, utilizing the Lemperle WAS to analyze standardized clinical photographs of the neck. Furthermore, the assessment of treatment efficacy was also performed by participants and two plastic surgeons who were not part of the research study at 1 and 6 months after the last treatment, using the Global Aesthetic Improvement Score (GAIS). The GAIS is rated on a 5‐point scale ranging from −1 (worse) to 3 (very much improved) and with a score of “very much improved,” “much improved,” or “somewhat improved” indicating a clinically significant improvement. The GAIS responder rate or effective rate of treatment was determined by combining the very much improved rate, much improved rate, and somewhat improved rate. Any discrepancies among observers were resolved through discussion until consensus was reached.

#### 2.5.2 Analysis of Collagen and Elastin

A CBS skin analysis system (CBS, Wuhan Bose Electronic Co. Ltd., Wuhan, China) was employed to noninvasively skin elasticity, collagen content, and skin oil content. Optical images captured through a microscope were subsequently processed into negative film. Utilizing color gradation recognition and texture scanning of the optical spectrum at 445 nm, the CBS skin analysis system is grounded in statistical methodologies [13].

It is well‐established that dermal collagen is primarily composed of type I collagen, accounting for approximately 80%–85%, and type III collagen, comprising about 10%–15%. The CBS skin analysis system employs the 3D Negative Technique to examine collagen fibers. The signals detected in the dermis are primarily generated by collagen type I fibers, which can be attributed to their structural properties and spatial arrangement within tissues. These signals from type I collagen fibers are readily discernible by the CBS skin analysis system [14].

The collagen and elastin levels of the neck skin were measured at four locations using the CBS Skin Analysis system. These locations included points a and b, situated at the junction of the most prominent neck wrinkle and the bilateral anterior border of the sternocleidomastoid muscle, point c, positioned at the midpoint between point a and the neck midline, and point d located at the midpoint between point b and the neck midline (Figure 2A).

### 2.6 Statistics Analyses

All the statistical analyses were performed using SPSS 26.0 software (SPSS Inc., Chicago, IL). Data with a normal distribution were expressed as mean ± standard deviations (*x*¯ ± *s*), and differences among multiple groups were analyzed with one‐way ANOVA followed by LSD test. Nonnormally distributed data were expressed as medians (lower quartile, upper quartile), comparisons between two groups were using Wilcoxon rank sum, and comparisons between three groups were using Kruskal–Wallis test. A value of *p* < 0.05 was considered statistically significant.

## 3 Results

### 3.1 Demographics

Twenty‐five women underwent eligibility assessment, with 10 failing to meet the inclusion criteria. The remaining 15 women were included in the study, with five subjects randomized to PRP injection group, five to the microneedles combined with topical PRP application group, and five to the RF combined with PRP injection group. There were no statistically significant differences in age and BMI among the groups (*p* > 0.05). No subjects were withdrawn from the study after the randomization.

### 3.2 Neck Wrinkle Severity Improvement

**FIGURE 3:**
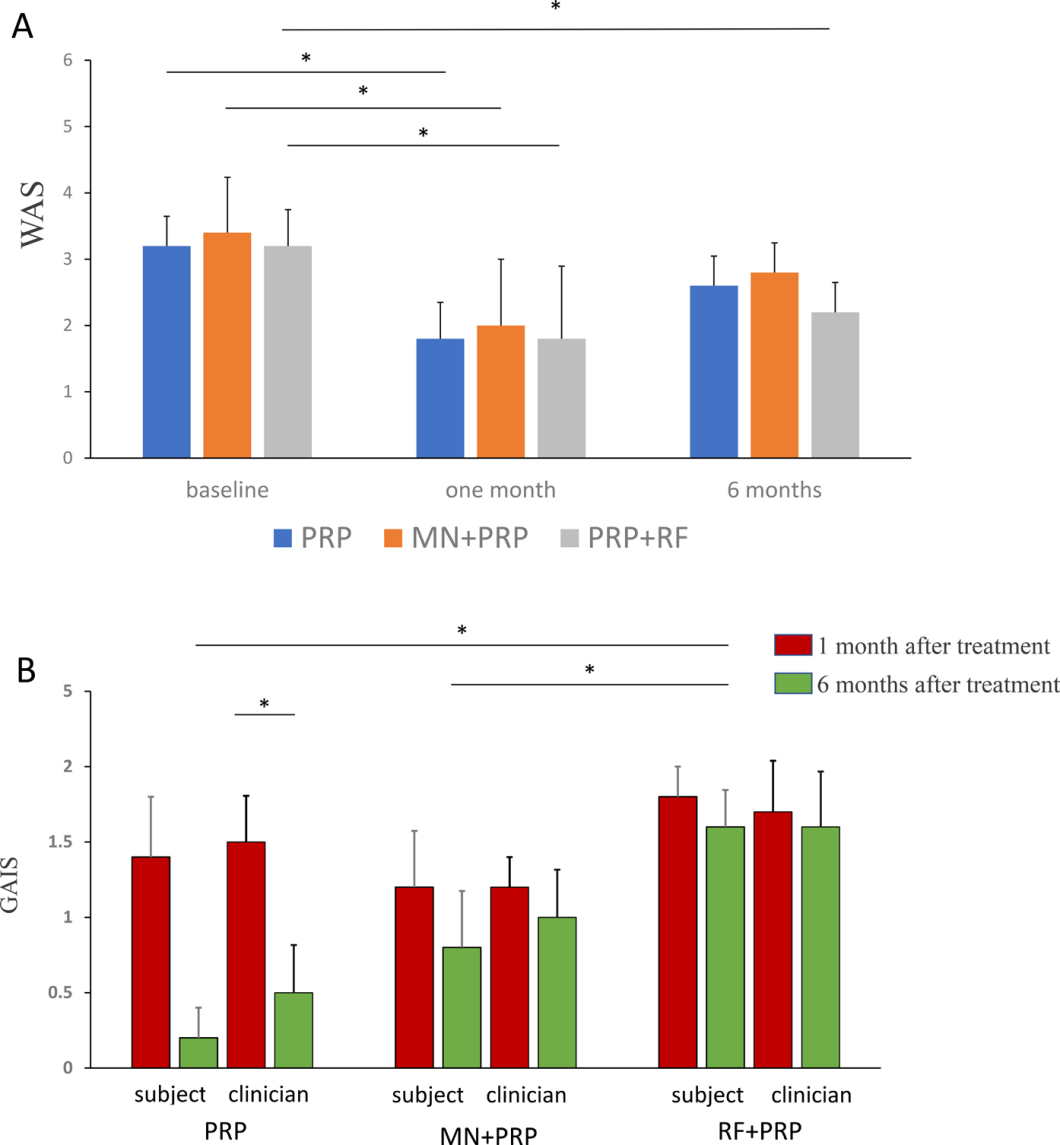
(A) Assessment of neck wrinkle by the observer using the Lemperle wrinkle assessment scale. *p < 0.05. (B) Global improvement scale scores assessed by investigators and subjects. *p < 0.05.

This study found that the WAS scores at both the 1‐month and 6‐month follow‐ups were lower than the pretreatment scores in all groups. Additionally, the scores at the 6‐month follow‐up were higher than those at the 1‐month follow‐up. Subjects in all groups demonstrated at least a one‐grade improvement in neck wrinkles based on WAS scores at the 1‐month follow‐up compared to baseline, with this improvement being statistically significant. At the 6‐month follow‐up, the WAS decreased in all three groups compared to baseline, with only the RF + PRP group showing a statistically significant decrease. There were no significant differences in the WAS among the groups at the 1‐month and 6‐month follow‐ups after treatment. (Figure 3A).

### 3.3 Global Aesthetic Improvement

One month after treatment, subjects in the PRP, MN + PRP, and RF + PRP groups reported GAIS responder rates of 80%, 80%, and 100%, respectively, while clinicians observed a 100% GAIS responder rate in all groups. There was no statistically significant difference in GAIS among the three groups (*p* > 0.05). Six months posttreatment, subject‐reported GAIS responder rates in the PRP, MN + PRP, and RF + PRP groups were 20%, 60%, and 100% respectively, with corresponding clinician‐reported rates of 40%, 80%, and 100%. The subject‐reported GAIS in the RF + PRP group was significantly higher than those in the other two groups (*p* < 0.05). Although clinician's GAIS was highest in group RF + PRP at 1 and 6 months after treatment, there was no statistical significance. (Figure 3B and Table 2).

**TABLE 2:** Global aesthetic improvement scale (GAIS) responder rate.

Group	Subject's self‐assessment		Clinician's assessment	
	1 month	6 months	1 month	6 months
PRP	80%	20%	100%	40%
MN + PRP	80%	60%	100%	80%
RF + PRP	100%	100%	100%	100%

### 3.4 Skin Elasticity and Collagen

**FIGURE 4:**
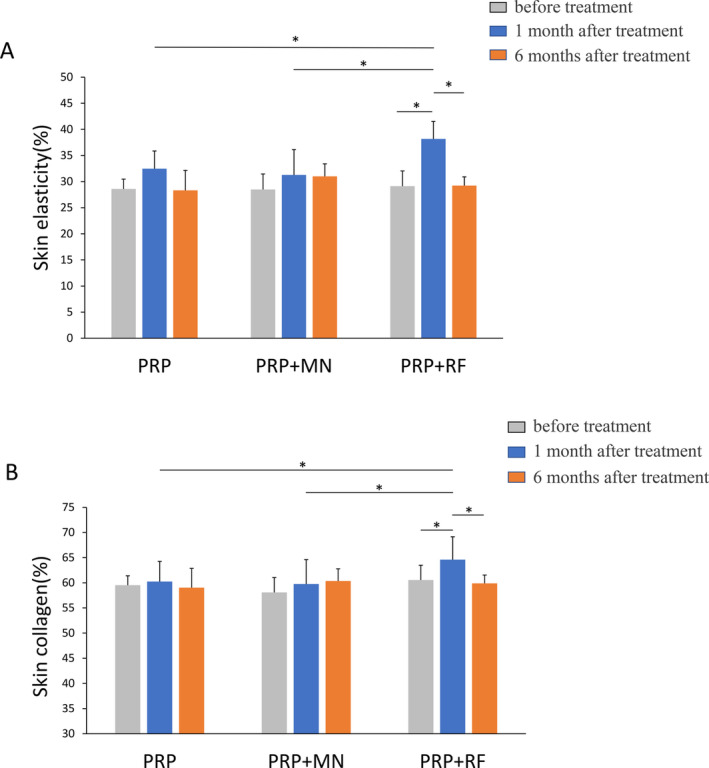
(A) Skin elastin as measured using CBS skin analysis. *p < 0.05. (B) Skin collagen as measured using CBS skin analysis. *p < 0.05.

There were no statistically significant differences in the mean values of skin collagen and elasticity at 1 and 6 months posttreatment compared to pretreatment in both the PRP and MN + PRP groups. However, the RF + PRP group exhibited a significant improvement in skin collagen and elasticity at 1 month posttreatment compared to pretreatment (*p* < 0.05), but not at 6 months posttreatment (Figure 4).

### 3.5 Side Effects

**FIGURE 5:**
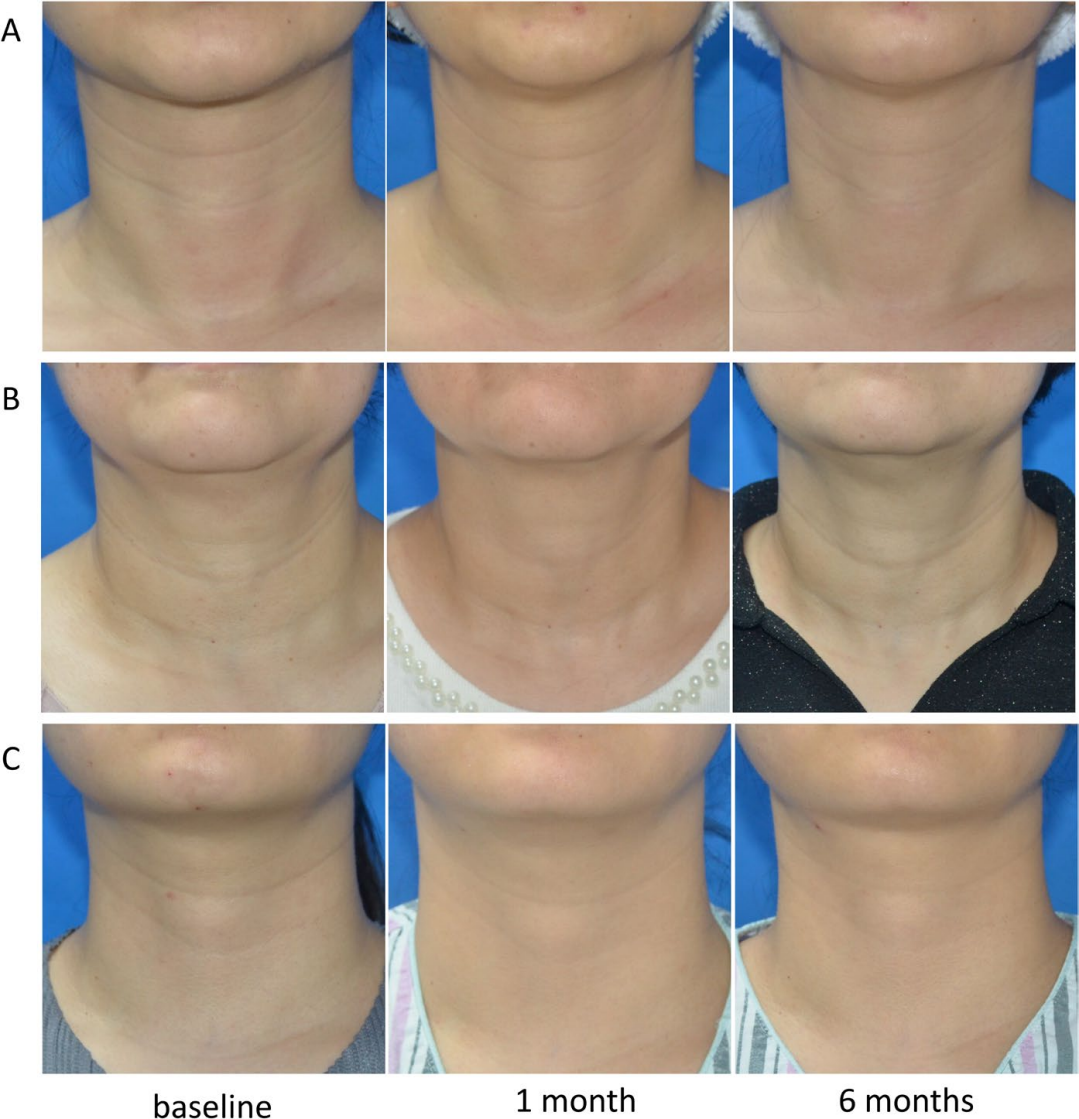
Clinical photographs of three representative patients in the three groups. (A) Patient in the PRP group at baseline and at two assessment time points following the last treatment (1 and 6 months). (B) Patient in the MN + PRP group at baseline, 1 and 6 months after last treatment. (C) Patient in the RF + PRP group at baseline, 1 and 6 months after last treatment.

No significant adverse effects were observed across all groups, with patients demonstrating tolerance to treatments without experiencing severe pain. Common local adverse effects, such as redness and swelling, these symptoms resolved within 2–7 days posttreatment. Some patients presented with ecchymoses at injection sites, which potentially attributed to the injection process or a higher concentration of red blood cells in the PRP. In the MN + PRP group, two subjects reported skin desquamation, while one subject displayed hyperpigmentation. Thermal discomfort of the neck skin increased immediately after RF treatment in the RF + PRP group and disappeared within 24 h (Figure 5).

## 4 Discussion

Neck wrinkles are one of the most prominent changes in neck aging and there are various reasons for its appearance, such as skin aging, photoaging, obesity, sagging cheeks, and the platysma pulls down the skin of the neck. To date, hyaluronic acid, autologous fat transplantation, and botulinum toxin A have been the most common methods for the treatment of neck wrinkles because of their convenience and effectiveness. Despite the well‐proven effectiveness of these treatments, some people prefer not to inject drugs or filler materials. Hence, PRP, RF, and MN are the best alternatives for wrinkle reduction.

PRP is an autologous biologic product with a high concentration of platelets, derived from centrifuged autologous blood. On activation by thrombin or Ca^2+^ ions, platelets from PRP release various growth factors, such as platelet‐derived growth factor (PDGF), transforming growth factor (TGF), epithelial growth factor (EGF), vascular endothelial growth factor (VEGF) and insulin‐like growth factor (ILGF), which are associated with wound healing, tissue regeneration, and repair [15]. According to a prospective controlled clinical study, PRP injection improved facial skin rejuvenation in 20 women by increasing dermal collagen production [16]. Therefore, PRP is widely used in many different medical fields and has been used by different methods such as local injection, topical application, and in combination with energy devices or MN [17].

In this study, we compared the effectiveness and safety of PRP application alone and combined with microneedles or RF in the treatment of neck wrinkles. WAS and GAIS assessments showed nearly all patients in our study observed improvements at 1 month after the last treatment which indicates that all the three treatments were effective in improving neck wrinkles at the 1‐month follow‐up. At 6 months after the last treatment, only a few patients exhibited improvement in the PRP group (20% improved for subjects and 40% improved for clinicians) while more than half of patients observed improvement in the MN + PRP group (60% improved for subjects and 80% improved for clinicians). Moreover, the PRP + RF group showed the best effects for the treatment of neck wrinkles and all patients still showed improvement at 6 months after the last treatment (100% improved for subjects and clinicians).

Clinical evidence suggests that PRP therapy is an effective treatment for aging skin [18, 19]. And numerous studies have proved that PRP monotherapy resulted in an increased number of fibroblasts, dermal collagen deposition, collagen density, epidermal, and dermal thickness [20, 21]. So we observed increases in both skin collagen and elasticity at 1 month after last treatment and PRP + RF group showed a statistically significant increase. But these increases in PRP group and MN + PRP group were not statistically significant, which might be due to the small sample size. Here we also found all these increases returned to baseline at 6 months after the last treatment. Therefore, there was an obvious decline in GAIS responder rates in PRP group and MN + PRP group, with the largest decrease found in PRP group. However, the GAIS responder rates did not decrease in the RF + PRP group. Wrinkle severity as assessed by LWAS also showed a significant reduction in the RF + PRP group. These suggest that the effect of combination treatments lasts for a long period of time, especially in the RF + PRP group.

The role of MN is to produce mechanical microinjuries on the skin to trigger tissue regeneration via inducing immune response and releasing cytokines. The result is the reduction of the wrinkles with increasing deposition of collagen and elastin. Furthermore, PRP therapy is combined with MN, serum is able to penetrate into the tiny openings in the skin and flow deeply beneath the surface [22]. RF differs from MN in that it uses the resistance of the skin tissue to convert RF energy into heat. The heat in the skin causes collagen contraction and new collagen formation [23]. In the present study, the patients in the combination therapy group showed better effectiveness at 6 months after last treatment. This could be attributed to the synergistic effects of MN and RF combined with PRP therapy.

The present study encompasses certain limitations. First, the relatively small sample size in each group may have compromised the statistical power to detect significant differences in the results, necessitating a reevaluation of the frequency of follow‐up. Second, the absence of biopsy or imaging techniques to evaluate histological changes in neck skin tissues pre and posttreatment presents a limitation. Third, the lack of a standardized protocol for PRP preparation and clear guidelines for the use of PRP, RF, and MN also pose limitations. Future research should endeavor to replicate these findings with larger sample sizes and the inclusion of a control group. Furthermore, it would be beneficial to compare various methods for treating neck wrinkles, such as MN RF and ultrasound technology. Subsequent investigations should prioritize standardizing PRP preparation methods and determining the optimal approach.

## 5 Conclusion

The application of PRP alone or in combination with MN or RF demonstrated varying degrees of clinical improvement, with the RF + PRP group exhibiting superior efficacy in treating neck wrinkles and skin laxity. These methods offer safe and effective treatment alternatives for patients seeking to avoid injections of drugs or filler materials injection.

## Author Contributions

Ke‐Cheng Li and Guang‐Hui Xie designed the experiments. Ke‐Cheng Li, Zi‐Zhe Lin, and Zhi‐Dan Zhang performed the research. Shan Xie analyzed the data. Guang‐Hui Xie supervised the research.

## Ethics Statement

This prospective, randomized, controlled study was approved by the Ethics Committee (KY‐2019‐043) and all procedures performed in studies involving human participants were in accordance with the ethical standards of the institutional and/or national research committee and with the 1964 Helsinki Declaration and its later amendments or comparable ethical standards. Written informed consent was obtained from all enrolled patients before the initiation of the study.

## Conflicts of Interest

The authors declare no conflicts of interest.

## Data Availability

The data that support the findings of this study are available on request from the corresponding author. The data are not publicly available due to privacy or ethical restrictions.
